# The Role of microRNAs in Ovarian Granulosa Cells in Health and Disease

**DOI:** 10.3389/fendo.2019.00174

**Published:** 2019-03-21

**Authors:** Jiajie Tu, Albert Hoi-Hung Cheung, Clement Leung-Kwok Chan, Wai-Yee Chan

**Affiliations:** ^1^Institute of Clinical Pharmacology, Anhui Medical University, Anhui, China; ^2^CUHK-SDU Joint Laboratory on Reproductive Genetics, School of Biomedical Sciences, The Chinese University of Hong Kong, Hong Kong, Hong Kong; ^3^Women's Health and Reproductive Medicine Centre, Hong Kong, Hong Kong

**Keywords:** miRNA, granulosa cells, PCOS (polycystic ovary syndrome), POF, GCT

## Abstract

The granulosa cell (GC) is a critical somatic component of the ovary. It is essential for follicle development by supporting the developing oocyte, proliferating and producing sex steroids and disparate growth factors. Knowledge of the GC's function in normal ovarian development and function, and reproductive disorders, such as polycystic ovary syndrome (PCOS) and premature ovarian failure (POF), is largely acquired through clinical studies and preclinical animal models. Recently, microRNAs have been recognized to play important regulatory roles in GC pathophysiology. Here, we examine the recent findings on the role of miRNAs in the GC, including four related signaling pathways (Transforming growth factor-β pathway, Follicle-stimulating hormones pathway, hormone-related miRNAs, Apoptosis-related pathways) and relevant diseases. Therefore, miRNAs appear to be important regulators of GC function in both physiological and pathological conditions. We suggest that targeting specific microRNAs is a potential therapeutic option for treating ovary-related diseases, such as PCOS, POF, and GCT.

## Introduction

The granulosa cell (GC) is a type of somatic cell arising from the sex cord in the ovary (1). During folliculogenesis, GCs develop from a thin, one-cell thick, layer around the oocyte in the primordial follicle to the multilayered cumulus oophorus surrounding the oocyte in the dominant follicle, that also has huge numbers of synthetically active mural GCs cells on its inner wall. The transition from primordial follicles to mature follicles involves differentiation and functional transformation of GCs. The interaction between GCs and oocytes is critical for coordinated oocyte maturation (2). Therefore, investigating the molecular pathways involved in proliferation, differentiation and functional transformation of GCs will lay a solid foundation for the mechanistic understanding of folliculogenesis (3).

GCs not only play a critical role in normal folliculogenesis, but also in pathological folliculogenesis in both benign disorders, such as polycystic ovary syndrome (PCOS) (4), premature ovarian failure (POF), which is also referred to as premature ovarian insufficiency, (POI) (5) and malignant disease such as ovarian GC tumors (GCT) (6). PCOS is a complex, multifactorial endocrine disorder affecting ~10% of all women of reproductive age (7). It is associated with multiple small antral follicles within the ovary that fail to develop into larger dominant follicles. GC function is different in polycystic ovaries. Increased follicle number and GC proliferation are observed in murine PCOS models (6). In addition, upregulated GC proliferation in smaller follicles is also observed in the ovaries of PCOS women (7, 8). Therefore, it is likely that abnormal ovarian GC proliferation has a role in the pathogenesis of PCOS. However, the underlying mechanism remains largely unclear. POF, with increased gonadotropin concentrations and hypoestrogenism, occurs in up to 4% of women under the age of 45 and it is associated with anovulation and infertility. The apoptosis of GC causes ovarian atresia and might eventually leads to POF. Therefore, the GC dysfunction is a main pathological feature of POF (8–12). The GC is the cell of origin in the pathogenesis of GCT (6), which is a clinically and molecularly unique subtype of ovarian cancer. GCT stems from the sex cord stromal cells of the ovary and represents ~5% of all ovarian cancers. While the etiologies of PCOS, POF, and GCT are unclear it is likely that both genetic and epigenetic factors may contribute to their pathogenesis (13–15), suggesting a role for differential gene function in these conditions. Genetic factors including, for example, critical genes for steroidogenesis and hormonal regulation and action, have been well-studied in these conditions. Additionally, in recent years, many noncoding portions of the genome have been recognized as being able to influence the epigenome with potential effects on the phenotypes of PCOS, POF, and GCT.

MicroRNA (miRNA) is a class of small noncoding RNAs (16). They are estimated to regulate mRNA translation of more than 70% of the protein-coding genes and are widely involved in both the normal and diseased states (17). Biogenesis of miRNAs is divided into several steps. Firstly, miRNAs genes are transcribed to primary miRNA (pri-miRNA) in the nucleus. Next, Drosha (a RNAase III enzyme) processes pri-miRNA to miRNA precursor (pre-miRNA). Following that, exportin-5 mediates the transport of pre-miRNA from nucleus to cytoplasm. Lastly, pre-miRNA is modified by Dicer to form mature miRNA, which forms the RNA-induced silencing complex (RISC) with Argonaute proteins. The complex searches for specific sequences of mRNA via the “seed sequence,” thus suppressing the translation or changing the stability of the target mRNAs (18). The role of miRNAs in normal development (19–22) and follicle pathogenesis (23, 24) has been summarized in previous reviews. Here, we focus on the recent findings regarding the roles of miRNAs in normal GCs and in the GCs of pathological conditions (PCOS, POF, and GCT). An in-depth understanding of miRNAs will provide valuable insights into the mechanism of normal GC development and the etiology and pathophysiology of these diseases with different GC phenotypes. In addition, the limitations and persistent issues that necessitate further investigation in the related areas are also discussed.

## The Role of Dicer in GC

As mentioned above, Dicer is an evolutionarily conserved ribonuclease III that is necessary for miRNA biogenesis. However, the specific functions of Dicer in the GC of the female reproductive system are unknown. Therefore, investigating the loss-of-function of Dicer in GC provides insight into the role of miRNAs in GC.

Otsuka et al. (25) firstly demonstrated that reduction of Dicer expression by a hypomorphic mutation causes infertility due to defects in ovarian angiogenesis and corpus luteum insufficiency. The terminal differentiation of GCs into luteinized GCs is known to regulate ovarian angiogenesis and normal corpus luteum function. In addition, *in vivo* ectopic expression of two microRNAs (miR-17-5p and let-7b) allows partial vascular recovery in the corpus luteum of these Dicer hypomorphic mice, suggesting that these effects are indeed miRNA-dependent. Other ovarian functions, including folliculogenesis, oocyte maturation, and ovulation, are not affected (25). However, this mouse model is not a GC specific knockout of Dicer, just a global effect of Dicer deficiency.

A possible role for miRNAs in the ovary is demonstrated by conditional knockout (cKO) of Dicer 1 in GC using the anti-Müllerian hormone (AMH) receptor type 2 promoter-driven expression of Cre recombinase (26). Therefore, two groups used this GC specific knockout of Dicer model and both showed that Dicer knockout in GC lead to female sterility. Nagaraja et al. (27) found that Dicer knockout in GC not only cause female sterility, but also induce multiple reproductive defects including decreased ovulation rates, compromised oocyte and embryo integrity, prominent bilateral paratubal cysts, and shorter uterine horns. MiRNA sequencing revealed differential expression of specific miRNAs in Dicer cKO mice. The majority of these miRNAs are predicted to regulate genes important for Mullerian duct differentiation and mesenchyme-derived structures, and several of these putative target genes were greatly affected upon Dicer cKO (27).

Second group also showed that adult female GC-specific Dicer KO mice display female sterility. Morphological and histological assessments of the reproductive tracts of immature and adult mice indicated that the uterus and oviduct were hypotrophic, and the oviduct was highly disorganized. Oviductal transport was disrupted in the GC-specific Dicer KO mice as evidenced by the failure of embryos to enter the uterus. These studies implicate Dicer/miRNA mediated posttranscriptional gene regulation in reproductive somatic tissues as critical for the normal development and function of these tissues and for female fertility (28).

Another group also used this GC specific knockout of Dicer mouse, but they focused on the regulatory role of Dicer in folliculogenesis. Lei et al. (29) demonstrated that the specific deletion of Dicer in GC led to an increased primordial follicle pool endowment, accelerated early follicle recruitment and resulted in more degenerate follicles. In addition, significant differences were observed in the expression of some follicle development related genes between cKO and WT mouse ovaries, such as Amh, Inhba, Cyp17a1, Cyp19a1, Zps, Gdf9, and Bmp15. With the Dicer inactivation, miR-503, a miRNA that is more abundant in ovary than in other tissues, was significantly decreased. Meanwhile, the expression of miR-503 decreased notably with follicle development in the gonadotropin-primed mouse ovary. Overexpression of miR-503 in primary GC resulted in the decreased expression of genes that related to GC proliferation and luteinization. Therefore, Dicer plays essential roles in follicular cell development through the differential regulation of expression of miRNA and target genes (29).

**Figure 1:**
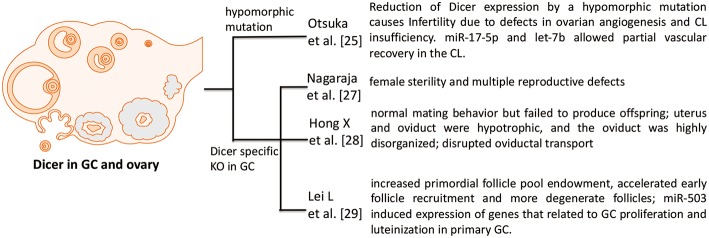
Phenotypes in Dicer KO ovary. Four studies investigate the role of Dicer in GC and ovary. Otsuka et al. (25) use a Dicer hypomorphic mutation mouse. Other three papers [Nagaraja et al. (27), Hong et al. (28), and Lei et al. (29)] show that specific deletion of Dicer in GC hampers the normal function and development of folliculogenesis in ovary.

These studies vary but overall show that Dicer in GC is fundamental for normal ovarian function and female fertility. Since Dicer is responsible for the synthesis of mature miRNAs, it is important to identify the key miRNAs that are essential for GC function (Figure 1).

## Physiological Conditions

### Transforming Growth Factor (TGF)-β (TGFB) Pathway

**Figure 2:**
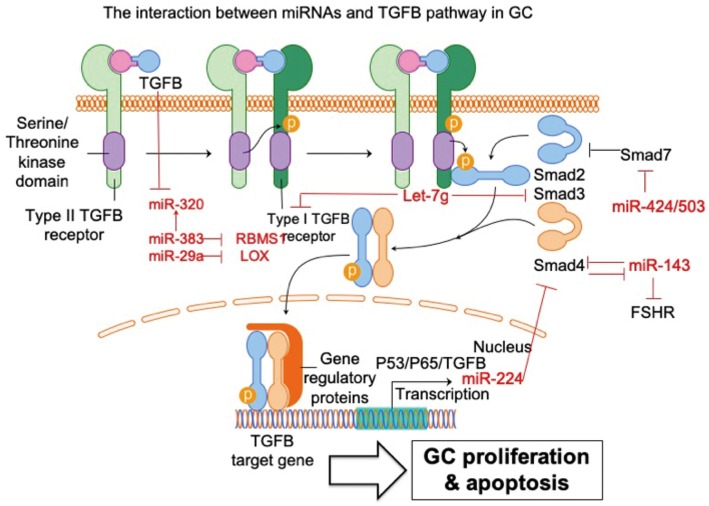
The interaction between miRNAs and TGFB pathway in GC. The interaction of miRNA-TGFB pathway in GC mainly affects the growth and survival of GC in ovary. FSHR, follicle-stimulating hormone receptor; SMAD3, SMAD family member 3; SMAD4, SMAD family member 4; SMAD7, SMAD family member 7; LOX, lysyl oxidase; RBMS1, RNA-binding morif single-stranded-interacting protein 1. Arrowhead line, promotion; Flat-end line, repression.

TGFB signaling plays an important role in reproduction. TGFB receptor 1 and 2 (types I and II) are membrane-bound serine/threonine kinase receptors that upon complexing with TGFB, activate SMAD 2/3 intracellular signaling by phosphorylation. Phosphorylated SMAD2/3 binds SMAD4 and translocates to the nucleus to regulate transcription of many downstream genes. Up-regulation of TGFB signaling represses GC apoptosis. In contrast, repressed TGFB signaling induces GC apoptosis, suggesting that TGFB signaling regulates GC survival (30, 31). However, the mechanism underlying TGFB signaling for GC survival/apoptosis has not been fully elucidated. TGFB pathway regulates a bunch of miRNAs in GC (32). In addition, emerging evidence shows that miRNAs might play a role in the regulation of the TGFB signaling pathway (Figure 2). The interaction of miRNA-TGFB pathway in GC seems to mainly dysregulate the normal growth of GCs.

Du et al. (33) show that the TGFB pathway controls ovarian porcine GC apoptosis through the SMAD4/miR-143 axis. Specifically, miR-143 enhances porcine GC apoptosis by regulating two direct targets, the FSH receptor (FSHR) and SMAD4. SMAD4 binds to the miR-143 promoter to form a feedback loop between the TGFB pathway and miR-143. Activated TGFB signaling rescues miR-143-reduced FSHR and intracellular signaling molecules, and miR-143-induced GC apoptosis. MiRNA-424/503 cluster members are involved in modulating bovine GC proliferation and cell cycle progression through targeting SMAD7 (a blocker of TGFB pathway) and consequently inducing phosphorylation of SMAD2/3 in GC (34). TGFB1 induces lysyl oxidase expression via repressing miR-29a, and lysyl oxidase is proven to be a direct target of miR-29a in human GCs (35). MiRNA let-7g promotes porcine GC apoptosis by targeting the TGFB1 receptor and repressing phospho-SMAD3. Therefore, let-7g blocks the TGFB pathway in GC (31). In addition, TGFB1 represses miR-383, which consequently inhibits another miRNA (miR-320) in murine GCs (36). The same research group further validated miR-383 as a downstream target of the TGFB pathway via direct repression of RNA-binding morif single-stranded-interacting protein (RBMS) 1 (37). The relationship between TGFB and miR-224 has also been investigated. Yao et al. (38) demonstrate that ectopic expression of miR-224 stimulates TGFB1-induced mouse GC proliferation through targeting SMAD4. This is the first demonstration that miRNAs could control reproductive functions by promoting TGFB1-induced GC proliferation. The same group further demonstrated that transcriptional cooperation between p53, p65, and TGFB regulates miRNA-224 transcription in murine GCs (39). Taken together, these studies support and further expands the knowledge of the powerful interaction between miRNAs and TGFB pathways in GC. It is reasonable to conclude that these miRNAs potentially regulate some essential cellular feature of GC, such as proliferation and apoptosis, via communication with the TGFB signaling pathway.

### Follicle-Stimulating Hormone (FSH) Pathway

**Figure 3:**
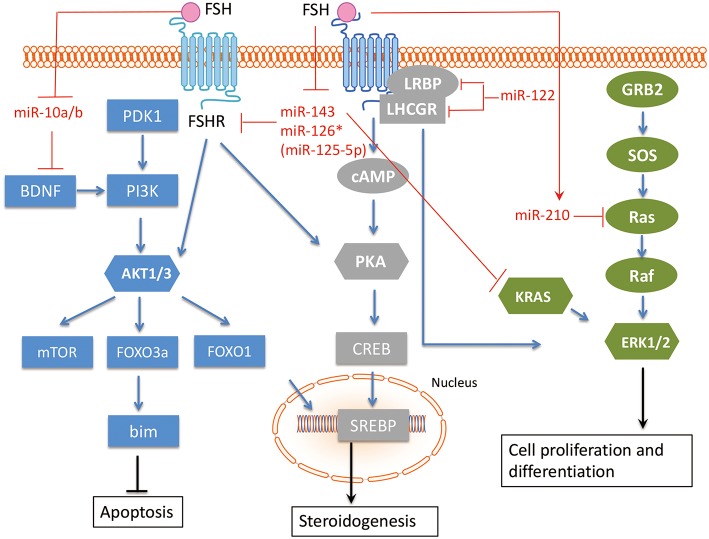
The interaction between miRNAs and FSH pathway in GC. The related miRNAs regulate GC proliferation, angiogenesis, hormone secretion and differentiation via interacting with FSH pathway in GC. FSH, Follicle-stimulating hormone; FSHR, Follicle-stimulating hormone receptor; BDNF, Brain-derived neurotrophic factor; KRAS, V-Ki-Ras2 Kirsten Rat Sarcoma 2 Viral Oncogene Homolog; Ras, rat sarcoma viral oncogene homolog; LHCGR, luteinizing hormone/chorionic gonadotropin receptor; LRBP, luteinizing hormone receptor mRNA-binding protein. Arrowhead line, promotion; Flat-end line, repression.

In ovaries, Follicle-stimulating hormone receptor (FSHR) is exclusively expressed in GCs (40). It is essential for their proliferation, growth, function, and differentiation (41). The ovarian follicle is characterized by two layers of GCs with different features; the cumulus GCs surrounding the oocyte and the mural GCs peripheral to the antrum (42). Treatment with FSH can induce the expression of more than 100 genes in GCs (43–46). FSH-induced regulation of the TGFB pathway has been demonstrated in GCs of different mammalian species, such as humans (47), mice (48), rats (49), and cows (50). On the other hand, TGFB signaling cooperates with Forkhead box L2 (FOXL2) to regulate FSHR and consequently affects GC functions, such as normal proliferation and differentiation, in pre-hierarchical follicles. During folliculogenesis FOXL2 plays a bidirectional modulating role involving intracellular FSHR transcription and GC proliferation via an autocrine regulatory mechanism, in a positive or negative manner, in cooperation with members of the TGFB superfamily; activin A (and its binding protein, follistatin) and GDF9 (51). MiRNAs can regulate GC proliferation, angiogenesis, hormone secretion and differentiation via interacting with the FSH pathway in GCs (Figure 3).

Shukla et al. (52) showed that FSH induced miR-210 expression in GC, and miRNA-210 consequently regulates pre-ovulatory GC proliferation and angiogenesis by repressing H-Ras and ephrin-A3. Zhang et al. (53) demonstrated that the FSH/miR-143/KRAS regulatory axis pathway plays an important role in regulating proliferation and estradiol secretion in murine GCs. In addition, the TGFB and FSH pathways interact via the miR-143/FSHR axis (33), highlighting the central role of miR-143 in GCs. Our group has found that two members in miR-10 family, miR-10a and miR-10b, are repressed by FSH in human, mouse, and rat GCs (54), suggesting a conserved role of miR-10 family in GC function. Indeed, we showed that both miR-10a and miR-10b could disturb normal development of GCs and folliculogenesis in different species. Overexpression of androgen receptors (AR) in GCs increased miR-126^*^ (miR-126^*^ is an old designation, it is designated as miR-125-5p now) and decreased FSHR expression. The FSHR gene is a direct target of miR-126^*^, which inhibits FSHR expression and increases the rate of AR-induced apoptosis. Therefore, AR and miR-126^*^ may cooperate to inhibit FSHR expression and induce apoptosis in GC (55). As GCs differentiate, they also express the luteinizing hormone/chorionic gonadotropin receptor (LHCGR). The regulatory role of miR-122 in FSH-induced LHCGR expression during follicle development was examined by Menon et al. (56). MiR-122 could completely abrogate FSH-mediated upregulation of LHCGR. MiR-122 also blocks the FSH-induced decrease in luteinizing hormone receptor mRNA-binding protein (LRBP) expression and increases the binding of LHCGR mRNA to LRBP. A transcription factor, Sterol Regulatory Element Binding Protein (SREBP-1a and SREBP-2 isoforms), is an intermediate in miR-122-mediated LHCGR mRNA regulation (57, 58). Therefore, miR-122 plays a regulatory role in LH/hCG-induced LHCGR mRNA downregulation by increasing LRBP expression through the activation of SREBP pathway.

FSH pathway has been implicated in modulating several key cellular functions of GC, such as proliferation, differentiation, and steroidogenesis, which are essential for the female reproductive system. Related studies have proven that these miRNAs are under control of FSH pathway in GC, including miR-10a/b, miR-143, miR-126^*^, miR-122, and miR-210. The miRNA-FSH pathway network in GC, especially the interaction among different miRNAs, should be further studied in the future. The interaction between above-mentioned miRNAs and the FSH pathway in GC is summarized in Figure 3.

### Hormone-Related Pathways

The differentiation process from primordial germ cells to fertilizable oocytes takes place within follicles (59). During this process, the key proteins required for oocyte maturation are progressively synthesized (60). This complicated follicular development is precisely modulated by different signals from different cells, including oocyte and the surrounding somatic GCs (61), in addition to the complex interactions between gonadotropin hormones, sex steroids, and other diverse growth factors (62). During folliculogenesis, these hormones, steroids, and growth factors produced by GCs also affect the process of follicular growth and GC development and function (63). Their differential effects on folliculogenesis is likely to be mediated by GCs and involve changes in GC proliferation, apoptosis, and hormone secretion (64). Here, we summarize the miRNAs that affect hormone secretion in GCs (Table 1).

**Table 1:** GC secretes hormones related miRNAs.

**miRNA**	**Hormones**	**Regulation**	**Target**	**Species**	**References**
miR-144	Prostaglan din E2 (PGE2)	Suppression	COX-2	Mouse	(65)
miR-375	Estradiol	Suppression	CRH	Porcine	(66)
miR-96	Steroid	Promotion	FOXO1	Human	(67)
miR-764	Estradiol	Suppression	SF-1	Mouse	(68)
miR-378	Estradiol	Suppression	Aromatase	Porcine	(69)
miR-320	Steroid	Promotion	E2F1 and SF-1	Mouse	(36)
miR-133b	Estradiol	Promotion	FOXL2	Mouse/human	(70)
miR-224	Estradiol	Suppression	SMAD4	Mouse	(39)
miR-383	Estradiol	Promotion	RBMS1	Mouse	(37)

MiR-144 represses prostaglandin E2, an important intrafollicular paracrine regulator with particular roles during ovulation, by inhibiting cyclooxygenase-2 expression (65). The corticotropin-releasing hormone signaling system is involved in numerous stress-related physiological and pathological responses, including inhibiting estradiol synthesis and follicular development in the ovary. MiR-375 mediates the corticotropin-releasing hormone signaling pathway and inhibits follicular estradiol synthesis (66). MiR-96 targets Forkhead box protein O1 (FOXO1) to induce steroid production in human GCs (67). MiR-764-3p regulates 17β-estradiol synthesis by repressing an essential transcriptional factor, steroidogenic factor-1 (SF-1), in mouse GCs (68). MiR-378 regulates ovarian estradiol production by targeting aromatase in porcine GCs (69). MiR-320 regulates steroid production by targeting E2F1 and SF-1 during follicular development (36). MiRNA-133b stimulates ovarian estradiol synthesis by targeting Foxl2 (70). MiR-224 is involved in TGFB1-mediated GC growth and estradiol production by targeting SMAD4 (39). Transactivation of miRNA-383 by SF-1 promotes estradiol release from mouse GCs by targeting RBMS1 (37). As mentioned before, several GC related hormones are essential for normal folliculogenesis and other associated diseases. Therefore, these miRNAs require more of our attention as they could regulate the secretion of related hormones.

GC is the main resource for synthesis and production of related hormones, such as prostaglandin E2, estradiol, steroid, for local use and providing endocrine signaling to other cells within ovary. Therefore, to investigate the role of those miRNAs that could regulate hormones synthesis in GC is essential for a lot of important functions in ovary, including follicular development, cumulus cell expansion, luteinization, ultimately oocyte maturation, and ovulation.

### Apoptosis-Related Pathways

**Figure 4:**
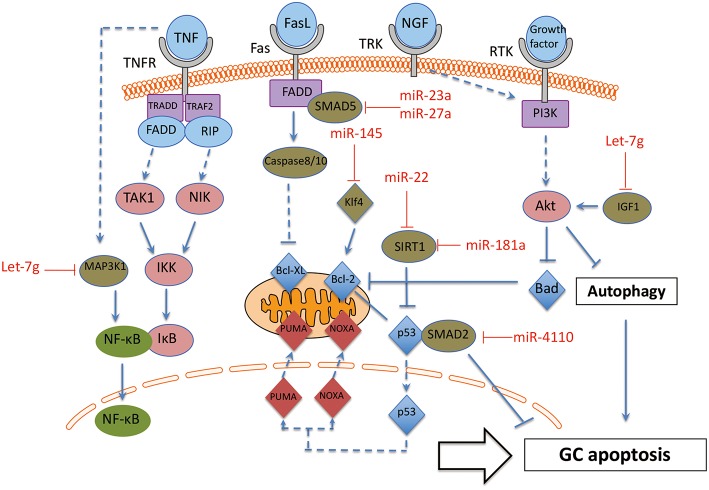
The interaction between miRNAs and apoptosis pathway in GC. These miRNA-mRNA communications could greatly modulate GC apoptosis during the process of follicle atresia. MAP3K1, Mitogen-activated protein kinase 1; Klf4, Kruppel-like factor 4; SMAD2, SMAD family member 2; SMAD5, SMAD family member 5; SIRT1, NAD-dependent deacetylase sirtuin-1; IGF1, insulin like growth factor 1. Arrowhead line, promotion; Flat-end line, repression.

Ovarian follicle atresia is mediated via apoptosis in higher vertebrates (71, 72). In most instances, GC are the first group of cells to show the features of apoptosis. Although follicle atresia is often considered as a normal physiological function to ensure the highest chance for ovulation of an appropriate number of healthy fertilizable oocytes, exceptional atresia leads to chronic infertility or menopause (73). Most research to investigate the molecular mechanism of GC apoptosis have been conducted in preclinical mammalian model systems (74). A series of studies have been shown that several miRNAs play essential roles in GC apoptosis. TGFB pathway, known to be regulated by miRNAs, affects apoptosis in GCs and the associated miRNAs have been summarized previously. Apart from the TGFB pathway, miRNAs can regulate GC apoptosis by targeting several other genes (75). Evidence for miRNAs that regulate GC apoptosis is shown in Figure 4 and discussed below.

The function of the miRNAs on apoptosis of GC can be divided into two groups: positively regulated apoptotic miRNAs and negatively regulated apoptotic miRNAs. On one hand, several miRNAs have been proven as apoptosis promoter of GC. MiR-4110 induces GC apoptosis via targeting SMAD2, and the ratio of an apoptosis index, Bcl-2-associated X protein (BAX)/B-cell CLL/Lyphoma 2 (BCL2), is also up-regulated in miR-4110 overexpressing GCs. Thus, MiR-4110 promotes GC apoptosis by targeting SMAD2 in the ovary (76). MiR-23a and miR-27a promotes apoptosis in human GC by targeting SMAD5 in the Fas ligand (FasL)-Fas signaling pathway (77). Anti-apoptotic genes BCL-2 and myeloid cell leukemia sequence (MCL) 1 are repressed by let-7g in GCs. Let-7g induces the expression of FOXO1 in GCs and leads to nuclear accumulation of dephosphorylated FoxO1, as a consequence of repression of the expression of Mitogen-activated protein kinase 1 (MAP3K1) in GC. Let-7g has been shown to induce GC apoptosis by targeting MAP3K1 in the porcine ovary (78). Inducers of oxidative stress, hydrogen peroxide (H2O2) and 3-nitropropionic acid (NP), induced miR-181a expression in GC *in vitro* and *in vivo*. Ectopic and knockdown of miR-181a promoted and repressed GC apoptosis, respectively. SIRT1 was also identified as a direct target of miR-181a and mediated the effects of miR-181a on GC apoptosis (79).

On the other hand, there are also some miRNAs that protect GC from apoptosis. Some cytokines and growth factors, such as Tumor necrosis factor-α (TNF-α), Fas ligand (FasL) and nerve growth factor (NGF) are specific inducers of apoptosis by affecting oxidative stress in GC (80). GC-specific miR-145 over-expression attenuates apoptosis in the *in vivo* ovarian oxidative stress model promoted by targeting Kruppel-like factor 4 (KLF4). Therefore, MiR-145 protects GC against oxidative stress-induced apoptosis via repressing KLF4 (81). In addition, miR-22 increases during follicular atresia and suppresses GC apoptosis. Further investigation showed that miR-22 inhibits mouse ovarian GC apoptosis by targeting NAD-dependent deacetylase sirtuin-1 (SIRT1) (82).

In addition, preantral and antral follicles involve several cell death pathways. Antral follicular degeneration stem from GC apoptosis, while preantral follicular atresia is mainly initiated by upregulated GC autophagy (24, 83). A series of publications reported the essential role of miRNAs in modulating autophagy (84). However, there is only one publication about miRNA on GC autophagy until now (85). This recent report showed that let-7g induced autophagy facilitate mouse GC apoptosis by targeting insulin like growth factor 1 (IGF-1). Therefore, further investigation definitively should focus on the effect of miRNAs on GC autophagy.

Taken together, these miRNA-mRNA interactions modulate GC apoptosis during the process of follicle atresia. Specifically, more attention should be paid to the two groups of miRNAs (promoter or suppressor of GC apoptosis) in GC apoptotic related disorders, such as POF and PCOS.

## Pathological Conditions

### PCOS

**Figure 5:**
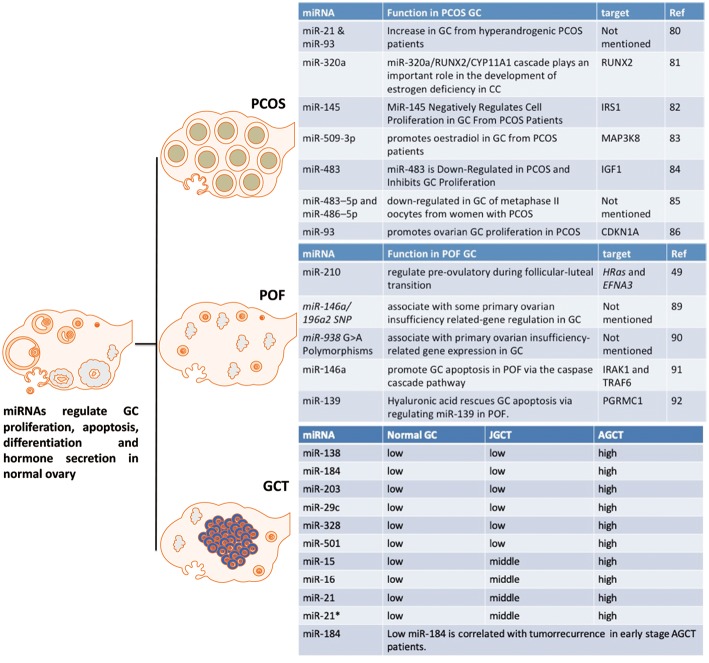
The effect of miRNAs on GC from PCOS, POF and GCT. These miRNAs are potential markers or targets for diagnostic, therapeutic, or prognostic treatment of PCOS, POF, or GCT.

The definition of PCOS is based on criteria from three different organizations: the National Institutes of Health (NIH) (86), the European Society of Human Reproduction and Embryology/American Society for Reproductive Medicine (Rotterdam) (87), and the Androgen Excess Society (88). The prevalence of PCOS in women of reproductive age varies from ~7% (NIH criteria) to up to 20% (the Rotterdam criteria) (7, 89). Polycystic ovaries, anovulation, and hyperandrogenism are the main features of PCOS according to all three criteria. In addition, PCOS is closely associated with many other pathological conditions, such as infertility, obesity, type 2 diabetes, dyslipidemia, insulin resistance and hypertension. There is evidence for differential GC function in PCOS and it is useful to summarize the differentially expressed functional miRNAs in GCs to determine potential therapeutic targets (Figure 5).

MiR-93 and miR-21 are increased in GCs from hyperandrogenic (HA) PCOS patients compared to Normo-androgenic (NA) patients. Free testosterone and free androgen index are positively correlated with of miR-93 and miR-21 in PCOS GCs. Androgens are fundamental in the pathophysiology of PCOS and androgens have effect on follicle growth, health and survival. As miR-93 and miR-21 have been highlighted as androgen responsive factors they may play a role in the follicular dysfunction involved in the pathogenesis of PCOS in hyperandrogenic condition (90). MiR-320a decreases in GCs from PCOS patients and this down-regulation is thought to cause relative estrogen deficiency. IGF1 regulated miR-320a in GCs, and miR-320a potentiates the steroidogenesis in CCs through modulation of cytochrome P450, family 11, subfamily a polypeptide 1 (CYP11A1) and cytochrome P450, family 19, subfamily a polypeptide 1 (CYP19A1), by directly targeting the osteogenic transcription factor Runt-related transcription factor 2 (RUNX2) (91).

Another miRNA, miR-27a-3p, also represses CYP19A1 via targeting cyclic AMP response element (CRE)-binding protein 1 (Creb1) in mouse GC from PCOS mice model. MiR-27a-3p induces apoptosis and decreases expressions of estradiol, aromatase and testosterone in mouse GC. The effects of miRN-27a-3p on the dysfunction of related hormones and apoptosis of GC could be involved in the PCOS pathophysiology (92). MiR-145 negatively regulates cell proliferation through targeting Insulin receptor substrate 1 (IRS1) in isolated ovarian GCs from PCOS patients. MiR-145 represses the activation of p38 mitogen-activated protein kinase (p38 MAPK) and extracellular signal-regulated kinase (ERK) and IRS1 rescues the suppressive effect of miR-145 on MAPK/ERK signaling pathways (93). “MiRNAome” and transcriptome in GCs from five PCOS and five control patients have been determined by a miRNA and cDNA microarray. The differentially expressed miRNA-509-3p and its potential target gene Mitogen-activated protein kinase 8 (MAP3K8) were identified from the miRNA and cDNA microarrays, respectively. MiRNA-509-3p promotes estradiol secretion by targeting MAP3K8 in GCs from PCOS patients (94). Repressed miR-483 expression is observed in the ovarian cortex from PCOS patients. MiR-483 is down-regulated in PCOS and inhibits GC proliferation via targeting IGF1 (95). MiR-483–5p and miR-486–5p are down-regulated in the cumulus GCs surrounding metaphase II oocytes taken from women with PCOS. MiR-486–5p promotes the proliferation of PCOS GCs via inducing PI3K/Akt pathway (96). MiR-93 expression is up-regulated in PCOS GCs and its predicted target, cyclin dependent kinase inhibitor 1A (CDKN1A), is repressed in PCOS GCs. MiR-93 induces GC proliferation and G1 to S transition. Inhibition of CDKN1A shows the similar cellular phenotype in GC. Therefore, miR-93 promotes the proliferation of ovarian GC through targeting CDKN1A in PCOS (97).

In summary, there is evidence for dysregulation of miRNAs in the GCs of women with PCOS with plausible actions that can be associated with the GC dysfunction in PCOS. That suggests that miRNA manipulation may have a plausible role in improving follicular health in women with PCOS. The above-mentioned miRNAs in GC may provide new insights into pathophysiology of PCOS.

### POF

POF is a reproductive disorder with significant health complications (98), including disorders of the genital tract, cognitive dysfunction, and cardiovascular related diseases (99). It is associated with a markedly reduced number of follicles in the ovary, which when present show some features consistent with GC dysfunction and it is likely that GC dysfunction can increase the likelihood of POF [6]. Therefore, summarizing miRNAs that could regulate GC dysfunction in POF may allow novel paradigms to improve GC health and slow the loss of follicles in POF patients (Figure 5).

The differential expression of miRNA-210 during the follicular-luteal transition regulates pre-ovulatory functions by targeting HRas and Ephrin A3 (EFNA3) (52). Single nucleotide polymorphisms of miR-146a/196a2, and their POI-related target genes, regulate GCs (100). In addition, there is an association of miR-938 G>A polymorphisms in GC with POI-related gene expression (101). MiR-146a has an important promoting effect on GC apoptosis by targeting interleukin-1 receptor associated kinase 1 (IRAK1) and tumor necrosis factor receptor-associated factor 6 (TRAF6) via the caspase cascade pathway (102). Hyaluronic acid is involved in promoting progesterone receptor membrane component 1 (PGRMC1) expression by regulating miR-139, which may be dysregulated in POF (103). Further studies are required for the potential application of these miRNAs as diagnostic, therapeutic, or prognostic markers of POF or even therapeutic targeting in the management of POF.

### GCT

Current histopathological and genetic markers, such as FOXL2 mutations, to distinguish between the two major subtypes, adult GCT (AGCT) and juvenile GCT (JGCT), are not accurate. The potential clinical utility of miRNAs as markers of GCT for tumor diagnosis and prognosis was evaluated by Cheng et al. (15). MiRNA-array results demonstrated that 37 miRNAs are differentially expressed between AGCT and JGCT. Six miRNAs, including miRs-138-5p,-184,-204-5p,-29c-3p,-328-3p, and -501-3p, were validated by RT-qPCR. Moreover, miR-184 was a potential predictor of tumor recurrence in AGCT, specifically for patients diagnosed with stage I and II and stage I only disease. It is the first report to profile miRNAome of human GCT. The role of these miRNAs in granulosa cell tumors is not yet understood and further studies are required to validate the clinical use of these miRNAs as diagnostic and recurrence markers as well as targets for manipulation.

## Conclusion and Prospects

This review describes miRNAs as a group of key posttranscriptional regulators in GCs of both physiological and pathological conditions. A single miRNA could repress hundreds, even thousands of genes, and a single gene could be modulated by multiple miRNAs. Many miRNAs are expressed in GC and directly regulate normal development and function of ovarian follicles (16), including atresia, ovulation, and ovarian steroidogenesis by targeting specific molecules and modulating various signaling pathways, such as TGFB-, FSH-, hormone-, and apoptosis-related pathways. In addition, miRNAs also play important roles by affecting GC in female reproductive diseases, such as PCOS, POF, and GCT. Systematically identifying miRNAs specific to GC will help researchers to better understand the underlying mechanisms relevant to ovarian disorders. For example, miR-320 affects steroidogenesis in both physiological (folliculogenesis) and pathological (PCOS) conditions of GC via targeting different targets. On one hand, during folliculogenesis, E2F1/SF-1 mediated miR-320-induced suppression of GC proliferation and of GC steroidogenesis; On the other hand, in PCOS patients, miR-320 decreases in GC and this down-regulation is thought to cause relative estrogen deficiency via targeting RUNX2, implying the potential therapeutic role of this miRNA in PCOS by regulating steroidogenesis. It may shed light on the essential regulatory roles of specific miRNAs in the development and function of the GCs, and follicles, paving the foundation for novel therapeutic strategies. Therefore, a deeper understanding of the regulation of GC function by miRNAs for treating ovarian diseases is definitely worth further investigation.

## Author Contributions

All authors listed have made a substantial, direct and intellectual contribution to the work, and approved it for publication.

### Conflict of Interest Statement

The authors declare that the research was conducted in the absence of any commercial or financial relationships that could be construed as a potential conflict of interest.
